# Co-delivery of Dacarbazine and All-Trans Retinoic Acid (ATRA) Using Lipid Nanoformulations for Synergistic Antitumor Efficacy Against Malignant Melanoma

**DOI:** 10.1186/s11671-020-3293-3

**Published:** 2020-05-19

**Authors:** Chenyang Li, Xiuping Han

**Affiliations:** Department of Dermatology, Shengjing Hospital of China Medical University, Shenyang, 110004 Liaoning China

**Keywords:** Melanoma, Dacarbazine, All-trans retinoic acid (ATRA), Lipid nanoformulations, Combination therapy

## Abstract

Malignant melanoma is a highly aggressive skin cancer responsible for 80% of mortality, and the overall median survival in patients with metastatic melanoma is only 6–9 months. Combination treatment through the simultaneous administration of dual drugs in a single nanocarrier has been demonstrated to be elegant and effective in combatting cancer. Herein, we employ a combination therapy based on dacarbazine (DBZ), FDA approved drug for melanoma and all-trans retinoic acid (ATRA), promising anticancer agents loaded on lipid nanoformulations (RD-LNF) as a new treatment strategy for malignant melanoma. We have successfully encapsulated both the drugs in lipid nanoformulations and showed a controlled release of payload over time. We demonstrated that the simultaneous delivery of DBZ and ATRA could effectively reduce cell proliferation in a concentration-dependent manner. The combinational nanoparticles significantly reduced the colony formation ability of B16F10 melanoma cells. Flow cytometer analysis showed that RD-LNF induced a greater proportion of apoptosis cells with significant inhibition of cell cycle progression and cell migration. These results suggest the promising potential of RD-LNF in the treatment of malignant melanoma with high efficacy.

## Introduction

Malignant melanoma is one of the aggressive malignancies occurs predominantly in the human skin [1]. Melanoma accounts for 4% of all dermatological cancers however it is responsible for 80% skin cancer mortality and becoming a growing concern in underdeveloped and developing countries [2, 3]. Melanoma has high tendencies to metastasize to the various parts of the body including the brain, heart, and lungs making it one of the aggressive forms of malignancies [4]. If diagnosed at early stages, surgical excision is on the potential therapeutic options. However, surgical intervention does not guarantee a complete recovery from melanoma [5]. Besides, radial growth phase of this malignancy becomes resistant to the majority of other treatment options including chemotherapy and radiotherapy [6]. To be specific, peptide receptors are overexpressed on the surface of melanoma cancer making it an attractive prospect. The subtype-2 somatostatin receptor (SSTR2) has been shown to highly express in the melanoma cells [7].

Dacarbazine (DBZ) or dimethyl-triazeno-imidazole carboxamide (DTIC), a DNA alkylating agent is the only and first-line choice of chemotherapeutic drug approved by the Food and Drug Administration (FDA) [8]. DBZ is a strong alkylating agent that kills the cancer cells by adding the alkyl groups to the DNA or nuclear materials of cancer cells [9]. Despite its potent action, DBZ suffers from poor aqueous solubility and short half-life (41 min) in blood circulation diminishing its therapeutic effect in the treatment of melanoma malignancies [10]. Besides, a disappointing response rate between 10 and 25% was observed with less than 5% complete cancer recovery indicating the limitation of a single agent therapy [11, 12]. Therefore, there is an urgent need to develop an alternative strategy to improve the treatment efficacy of DBZ.

Co-delivery of multiple therapeutic agents in single administration proven to be more effective compared to that of the individual therapeutic agent [13]. The co-delivery of therapeutic agents in a single carrier system will confer the synergistic activity, similar pharmacokinetic properties, and higher anticancer efficacy [14]. All-trans-retinoic acid (ATRA) is a promising anticancer agent used in the treatment of several cancers [15]. The ATRA exhibited its therapeutic efficacy by binding to the retinoic acid receptors in the nucleus of cancer cells resulting in the inhibition of growth, proliferation, differentiation, and eventual cell death [16]. Unlike other chemotherapeutic agents, ATRA did not result in any adverse effects such as cardiotoxicity or bone marrow hypoplasia. ATRA has been reported to enhance the anticancer efficacy when used in combination with an appropriate chemotherapeutic agent. Again, lipophilic ATRA suffers poor aqueous solubility and rapid systemic clearance necessitating the need for a stable delivery system [17].

Nanoparticulate delivery systems have been shown to improve the therapeutic efficacy of encapsulated therapeutics by releasing it in the target tumor tissues and by avoiding the off-target effect in the systemic circulation employing enhanced permeation and retention (EPR) effect [18, 19]. The solid lipid nanoparticles (SLN) are considered to be alternative to many existing carriers including liposomes or micelles due to the salient features such as enhanced stability, controlled drug release, high-loading efficiency, and ease of preparation/scale-up [20]. One of the important aspects of SLN as a drug delivery carrier is the safety profile of lipids which are of GRAS status, well-tolerated, and physiological lipids [21]. The stable incorporation of drugs in the lipid nucleus improves the aqueous solubility and improves the pharmacokinetic profile and extends the physiological stability in the systemic circulation [22].

In this study, we address a promising strategy of co-delivery of DBZ and ATRA using lipid nanoformulations for the combination treatment in melanoma malignancies. The DBZ is expected to load in the lipid core while ATRA is expected to be a part of the nanoparticle structure. We studied the physicochemical properties, cellular uptake, and in vitro cytotoxicity of combined nanoparticles. In addition, the anticancer effect was further evaluated by apoptosis assay, cell cycle analysis, colony formation, and cell migration analysis.

## Conclusion

In conclusion, we have successfully formulated dacarbazine and all-trans retinoic acid-loaded lipid nanoformulations. Our results clearly demonstrate that the RD-LNF inhibits melanoma cell proliferation, induces remarkable apoptosis, and inhibits cell cycle progression and cell migrations. Futuristic work will focus on studying the anticancer efficacy in clinically relevant animal models and developing a targeted therapy towards the melanoma malignancy. This is a preliminary study carried out in the melanoma cells and broad range studies in different clinically relevant animal models are the next part of our research work.

## Materials and Methods

### Preparation of DBZ/ATRA-Loaded Lipid Nanoformulations

The drug-loaded lipid nanoparticles were prepared by the sonication method. Briefly, 10 mg of DBZ and 10 mg of ATRA were dissolved in 2 ml of a chloroform solution containing 50 mg of Egg l-α-phosphatidylcholine (PC) and 2 mg of DSPE–methyl(polyethylene glycol)-2000 (mPEG_2000_). The organic solvent was dried using argon gas exposure for 20 min. The dried drug + lipid mixture was added with 80 mg of trimyristin (Tm) and incubated at 65 °C for 1 h. To this oil mixture, 5 ml of 4% poloxamer solution was added and immediately probe sonicated at 80 W for 6 min. The resulted emulsion was cooled at ice for 30 min. The nanoparticles were subjected to Amicon Ultra 0.5 centrifugal filter unit (3 kDa cutoff; Merck, Germany) at 12000×*g* for 20 min. The amount of free DBZ and ATRA in the filtrate was evaluated by HPLC method and loading efficiency and loading capacity were calculated. Waters HPLC system consisting of a Waters 1525 binary pump, Waters 2487 UV detector, Waters 2475 fluorescence detector, 1500 column heater, and a Symmetry C18 column was used for the drug analysis. For ATRA, the mobile phase consisted of acetonitrile and trifluoroacetic acid in 90/10 v/v ratio at a flow rate of 1 ml/min and detected at 348 nm. For DBZ, acetonitrile and 0.05 M disodium hydrogen phosphate in 30/70 v/v ratio containing 0.5% of TEA was used. The pH of the mobile phase was maintained at pH 3.7 and a flow rate of 1 ml/min was used.

### Particle Size and Morphology Analysis

The particle size distribution of combined nanoparticle was evaluated by Malvern Zetasizer Nano ZS90 with a He-Ne laser (633 nm). All samples were diluted with distilled water and the experiments were performed at 24 °C in triplicate. The morphology of the nanoparticle was evaluated by transmission electron microscope (TEM; JEOL JEM200CX at 120 kV). The diluted particles were stained with 2% phosphotungstic acid, dried, and observed under TEM.

### In Vitro Drug Release of Drug-Loaded Nanoformulations

The release of encapsulated drugs was evaluated by the dialysis method. Two milliliters of drug-loaded nanoparticles containing an equivalent of 1 mg of ATRA and 1 mg of DBZ was sealed in a dialysis membrane (Spectra/Por, MWCO 3.5 kDa). The dialysis tube was placed in a 25 ml of release buffer and kept at a rotation of 100 rpm at 37 °C. At predetermined time interval until 72 h, 1 ml of samples were withdrawn and replaced with fresh buffer of equal quantity. The amount of DBZ and ATRA was evaluated by HPLC method as described above.

### Cell Culture and Cellular Uptake Analysis

Murine skin melanoma cells (B16F10) were purchased from China Infrastructure of Cell Line Resources (Beijing, China). The cells were cultured in RPMI-1640 medium supplemented with 10% of FBS and 1% of the antibiotic mixture. Mediums were regularly changed every 2 days and cultured after 90% confluency. For cellular uptake analysis, B16F10 cells were seeded in a 6-well plate for 24 h incubation. The nanoparticles were loaded with Coumarin-6 as a fluorescent tracker. The old medium was removed and replaced with a fresh medium containing the Coumarin-6-lipid nanoparticles and incubated for 1–3 h in reverse order. The cells were washed and scrapped with cell scrapper. The cells were centrifuged at 1200 rpm for 5 min and the cell pellet was resuspended in 1 ml of cold PBS. The samples were assessed by AccuriTM C6 flow cytometer (BD Co., USA).

### In Vitro Cytotoxicity Assay

The cytotoxic effect of free ATRA, DBZ, D-LNF, and RD-LNF on B16F10 cell was evaluated by MTT assay. The cells (1 × 10^4^) were seeded in each well of the 96-well plate and incubated for 24 h. The cells were first treated with different concentrations of free ATRA and DBZ and tested its anticancer effect on the melanoma cells. Followed by, cells were treated with a fixed concentration of free ATRA, DBZ, D-LNF, and RD-LNF at 25 μg/ml and 50 μg/ml, respectively. The cells were incubated for 24 h and then the media was carefully removed and washed twice with PBS. At the end, cells were treated with 100 μl of 5 mg/ml MTT solution and incubated for 4 h. The culture medium was carefully removed and 100 μl of isopropanol was added and incubated for 15 min under shaking conditions. The dissolved formazan crystals were studied at 570 nm using a microplate reader. Non-treated cells were used as control and calculations were made based on the cell viability of controls.

### Apoptosis Assay—Flow Cytometer

The apoptosis effect of free ATRA, DBZ, D-LNF, and RD-LNF in B16F10 cells was evaluated following the staining with PE annexin V and 7AAD-based apoptosis kit. The cells were seeded in a 12-well plate at a cell density of 2 × 10^5^ cells/well and incubated for 24 h. The cells were treated with 25 μg/ml equivalents of free ATRA, DBZ, D-LNF, and RD-LNF formulations and untreated cells were considered as a control. After 24 h of treatment exposure, cells were stained as per the manufacturer’s protocol. AccuriTM C6 flow cytometer was used for PE and 7AAD expression and a minimum of 10,000 events were acquired in the flow cytometer. PE-positive and 7AAD-negative cells were early apoptotic; PE-positive and 7AAD-positive cells were late apoptotic.

### Cell Cycle Analysis—Flow Cytometer

The cells were seeded in a 12-well plate at a cell density of 2 × 10^5^ cells/well and incubated for 24 h. The cells were treated with 25 μg/ml equivalents of free ATRA, DBZ, D-LNF, and RD-LNF formulations and untreated cells were considered as a control. After 24 h, treated cells were washed and collected by trypsinization and fixed with 70% ethanol for 2 h at 4 °C. Cells were then treated with ribonuclease to get rid of any RNA contaminations. Now, the cells were stained with propidium iodide (PI) for 30 min at 37 °C in the incubator. The fluorescence of PI-stained cells was determined by the AccuriTM C6 flow cytometer. The cell cycle phase was divided into subG1, G1, S, and G2/M phase.

### Colony Formation Assay

The cells were seeded in 12-well plates at a cell density of 2 × 10^5^ cells/well and incubated for 24 h. The cells were treated with 25 μg/ml equivalents of free ATRA, DBZ, D-LNF, and RD-LNF formulations. The treated cells were trypsinized, washed and counted using a cell counter. The cells were then seeded in a 6-well plate at a density of 1500 cells/well. The cells were then incubated under ambient conditions of 37 °C for 12 days until the colonies are visible. The cells were washed with PBS and fixed with methanol to acetic acid to water (1:1:8) for 10 min, followed by staining with 0.1% crystal violet stain for 45 min. The stained cells were then observed under a light microscope.

### Cell Migration Assay

Twelve-well transwell chamber with 8 μm pore size was used for this assay. To initiate the study, 5 × 10^4^ cells/well was seeded in the upper chamber. The upper-medium is devoid of FBS while the lower chamber medium consists of 10% FBS. After 24 h, the amount of cells migrated to the lower membrane surface was fixed and stained with 0.5% crystal violet dye. The number of cells was counted in five fields that were randomly selected under a light microscope; the average number of cells was recorded and analyzed.

### Statistical Analysis

Data are presented as mean ± SD and performed in triplicate unless and otherwise mentioned separately. Two groups were compared using unpaired *t* tests, while the comparison of more than two groups was done using one-way ANOVA with Turkey’s multiple-comparison test. Statistics were performed using GraphPad Prism software and a difference of *p* < 0.05 was considered significant.

## Results and Discussion

### Preparation and Characterization of DBZ/ATRA-Loaded Lipid Nanoformulations

**Fig. 1:**
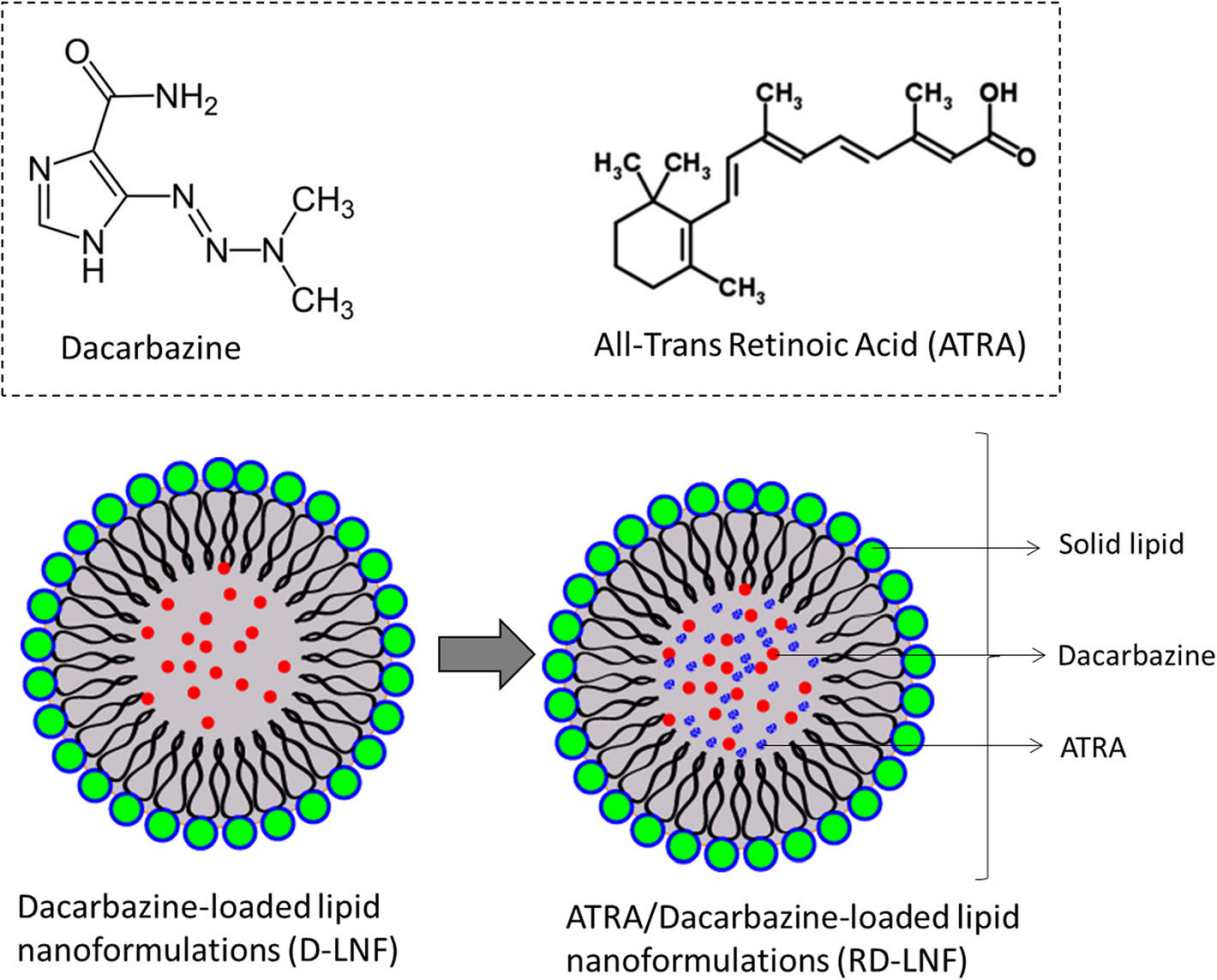
Structure of dacarbazine (DBZ) and all-trans retinoic acid (ATRA). Schematic illustration of DBZ/ATRA-loaded lipid nanoformulations is presented. The lipid nanoformulation was prepared by ultrasonication method. Both the DBZ and ATRA are hydrophobic molecules with a molecular weight of less than 300 g/mol. The hydrophobic molecules are expected to concentrate in the core of the nanoparticles stabilized by a surfactant

**Fig. 2:**
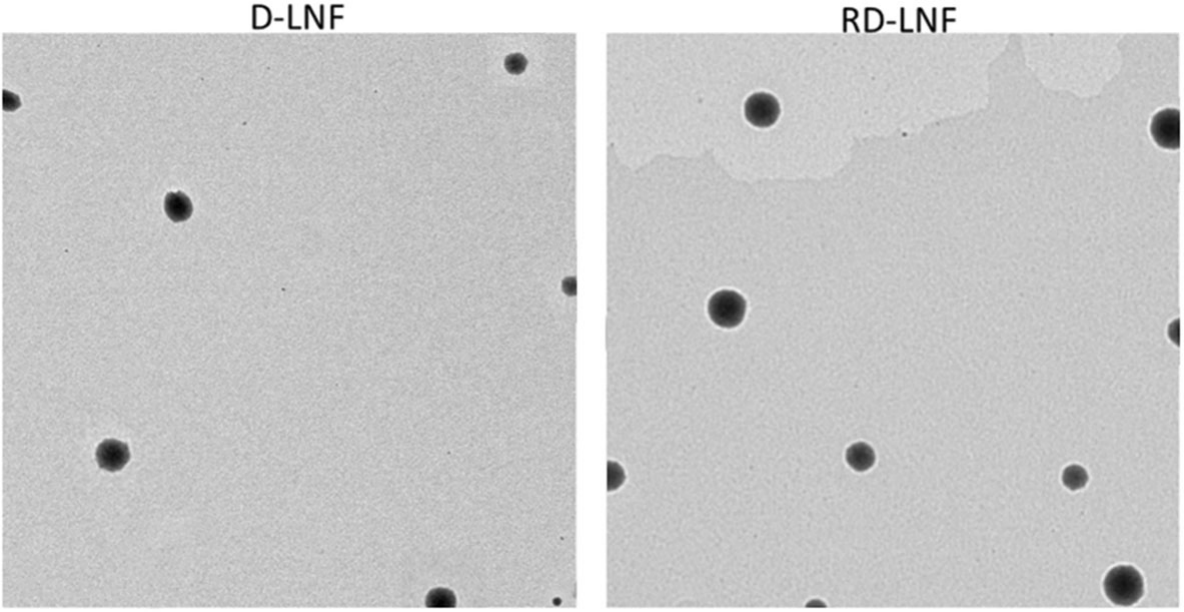
Morphological analysis of D-LNF and RD-LNF using transmission electron microscopy. Representative image depicting the accurate morphology is presented

The treatment of melanoma remains a major challenge and standard treatment options including chemotherapy or radiotherapy or surgical resection will be effective only on the solid bulk tumors accompanied by poor prognosis. FDA has approved drugs such as paclitaxel or its combination, but it failed to improve the overall impact on patient survival and cancer cure. Dacarbazine (DBZ) is the first-line choice of chemotherapeutic drug approved by FDA; however, DBZ suffers from poor physicochemical properties and resulted in a disappointing response rate between 10 and 25%. To deal with these challenges, we have developed a novel therapeutic strategy of combination with ATRA and DBZ in lipid nanoformulations (Fig. 1). We hypothesized that the combination of two therapeutic components will enhance the antitumor efficacy in melanoma malignancies. It is worth noting that ATRA though exhibits the anticancer properties, did not result in any adverse effect in the systemic environment [23]. As the DBZ is hydrophobic in nature, we have designed lipid nanoformulations that can stably incorporate the hydrophobic drugs in the lipid core and ATRA will be loaded as a structural component of the nanoparticles. The lipid nanoparticles carrying the therapeutic components will help deliver in the far deep tumor in the body. The loading efficiency of DBZ and ATRA was 91.2 ± 1.25% and 95.8 ± 1.14% respectively. The loading capacity of DBZ and ATRA in RD-LNF was 7.07 ± 0.65% w/w and 7.48 ± 1.05% w/w, respectively. The particle size of D-LNF was observed to be 121.5 ± 1.65 nm with a polydispersity index (PDI) of 0.134 and zeta potential of -23.5 ± 0.85 mV. The particle size of RD-LNF was 138.2 ± 1.28 nm with a PDI of 0.159 and zeta potential of − 25.4 ± 0.58 mV. The increase in overall particle size was mainly attributed to the structural loading of ATRA; however, the final size of RD-LNF was less than 150 nm allowing to preferentially accumulation the malignant melanoma tumors owing to the EPR effect. Besides, the presence of hydrophilic PEG will prevent the aggregation of particles and reduce its uptake by the reticuloendothelial system (RES) and thereby improve its systemic performance in the body. The surface charge around – 25 mV will confer excellent storage stability. The particle morphology was investigated by transmission electron microscope (TEM) (Fig. 2). As seen, both D-LNF and RD-LNF are perfectly spherical in size and distributed evenly in the copper grid. The difference in size between D-LNF and RD-LNF was consistent with the DLS observation. Lack of particle debris, small particles, and aggregation indicates the success of the formulation process.

### Stability Analysis and In Vitro Drug Release from D-LNF and RD-LNF

**Fig. 3:**
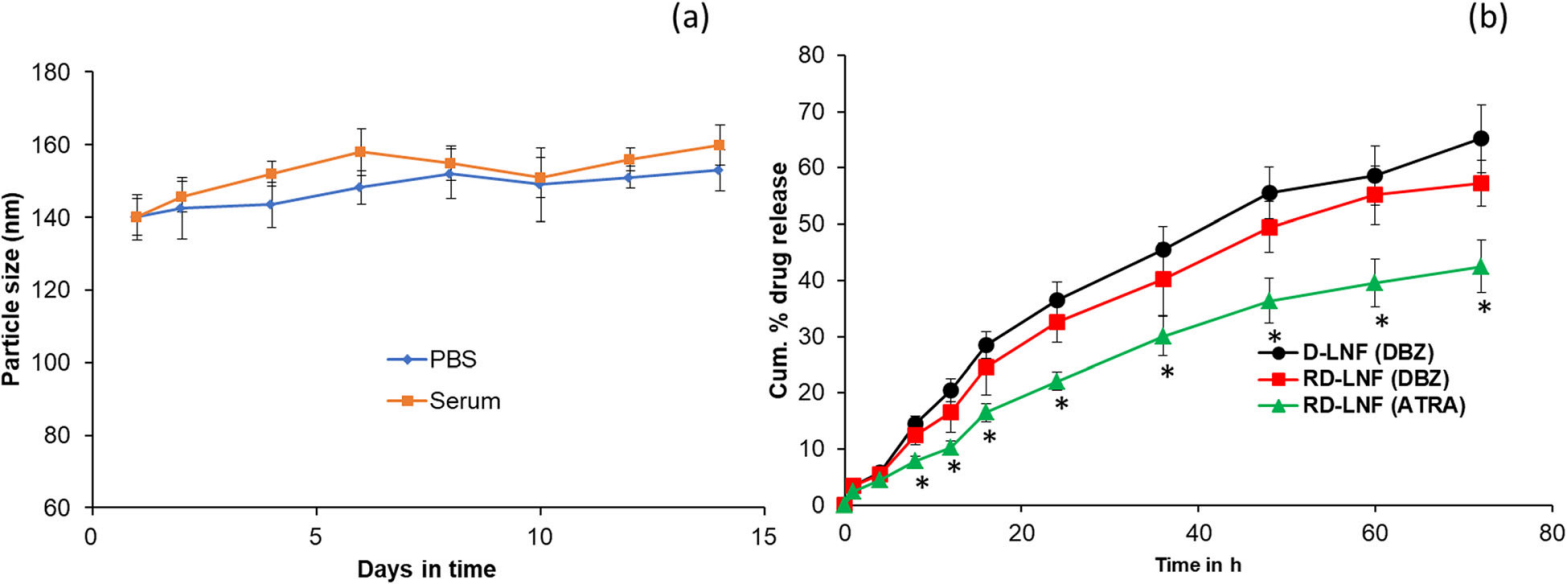
**a** Stability analysis of RD-LNF in pH 7.4 and serum (10% FBS) conditions. **b** In vitro drug release of DBZ from D-LNF; and in vitro drug release of DBZ and ATRA from RD-LNF. The release study was performed in phosphate-buffered saline (pH 7.4) for 72 h study period. Results are presented as mean ± standard deviations (*n* = 4)

The RD-LNF exhibited an excellent stability at both pH 7.4 (PBS) and serum (10% FBS) conditions (Fig. 3a). The particle size did not show any significant increase in both the conditions throughout the study period indicating the stability of nanoparticles. The ability of drug release in a timely manner decides the effectiveness of the drug-loaded nanoparticle systems. We have performed the release kinetics of DBZ and ATRA from D-LNF and RD-LNF in phosphate-buffered saline (PBS, pH 7.4). As shown in Fig. 3b, no burst release or phased release of drug was observed from either of the two-nanoparticle systems indicating the stable loading in the core of the nanoparticles and not in the nanoparticle surface. No significant difference in the release of DBZ was observed from D-LNF and RD-LNF indicating that the presence of ATRA did not slow or alter the drug release pattern. A slight delay in the drug release of DBZ from RD-LNF might be attributed to the increased path length attributed to the ATRA in the lipid structure. It is interesting to note that ATRA released significantly slower compared to that of DBZ in the RD-LNF carrier system. Significant differences were started to appear after 24 h until 72 h mainly attributed to the extreme hydrophobic nature of ATRA and being part of the structural component. Overall, lack of burst release and controlled release of therapeutic components will benefit the melanoma cancer treatment.

### In Vitro Cellular Uptake

**Fig. 4:**
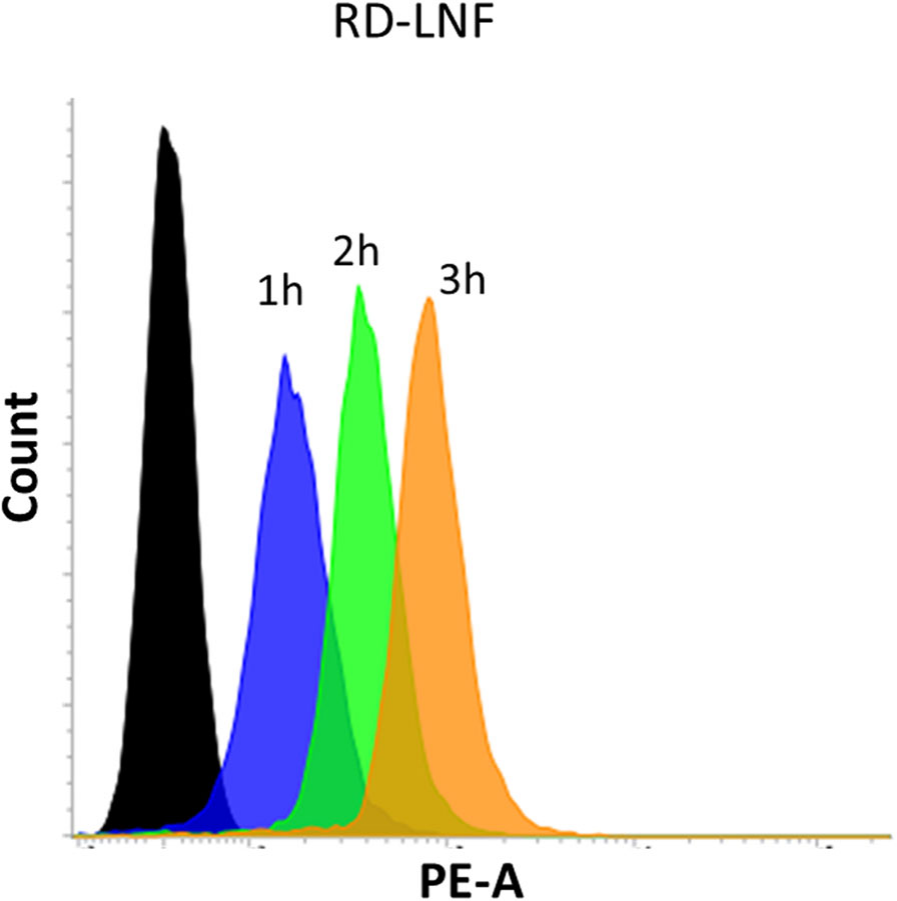
In vitro cellular uptake analysis of B16F10 melanoma cells. The cancer cells were treated with RD-LNF and incubation times were varied from 1h to 3 h and cellular uptake was analyzed using flow cytometer. The Coumarin-6 was used as a fluorescent tracker

Higher or enhanced cellular uptake of the nanoparticle determines the therapeutic efficacy in the cancer cells. We have performed the cellular uptake of RD-LNF in B16F10 cancer cells and Coumarin-6 was used as a fluorescent tracker. As shown in Fig. 4, remarkable internalization of nanoparticles was observed after 1 h of nanoparticle incubation and the uptake consistently increased until 3 h. The remarkable uptake of lipid nanoformulations is consistent with the higher cellular uptake of the lipid-based carrier system. It was speculated the passive diffusion and endocytosis-based mechanism might be responsible for the higher cellular uptake. The nanoparticles after the endocytosis will reach the lysosome where the encapsulated drugs are liberated and exhibit their respective pharmacological actions.

### In Vitro Cytotoxicity Assay

**Fig. 5:**
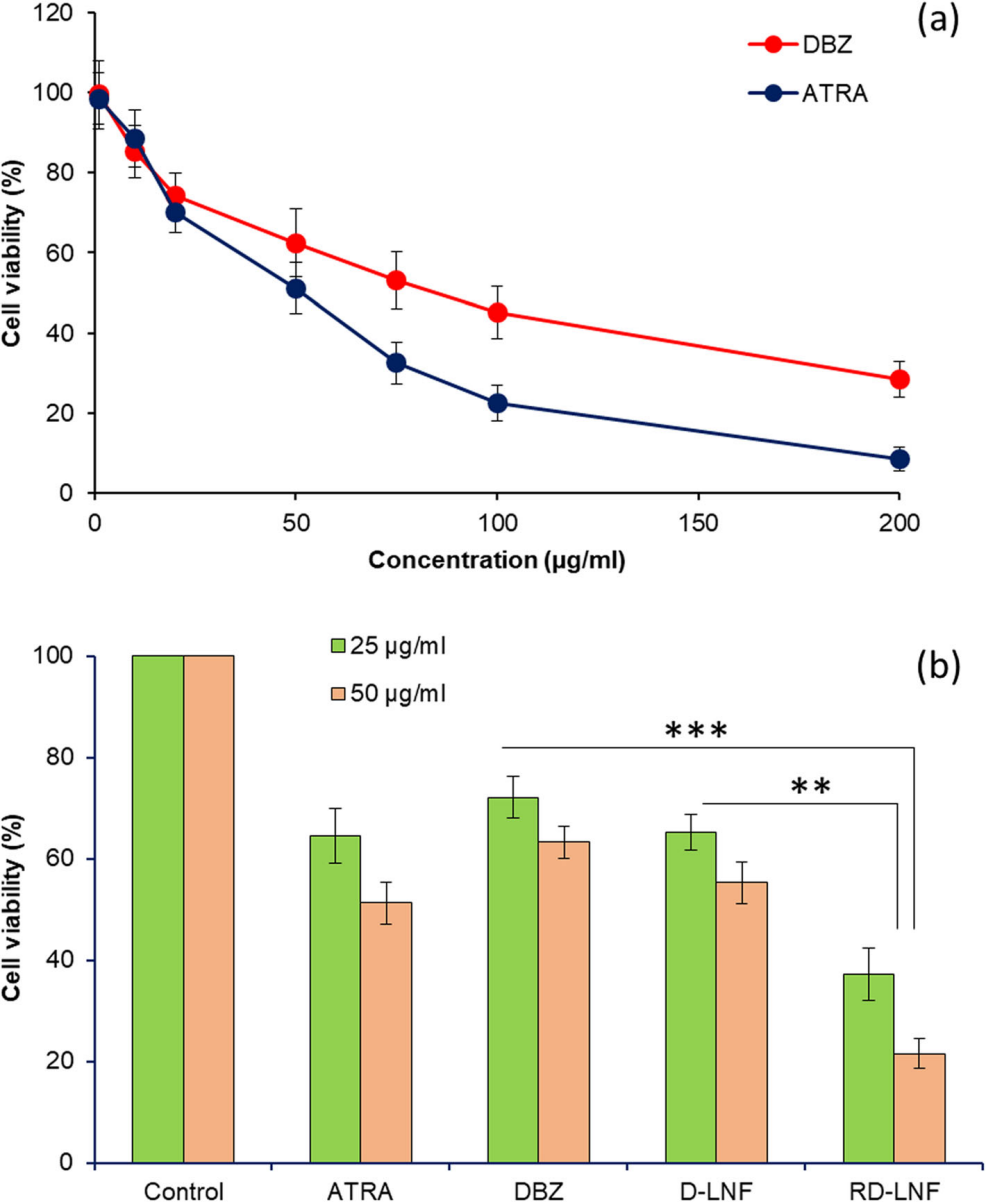
**a** In vitro cytotoxicity assay of free DBZ and ATRA in a concentration-dependent manner. **b** In vitro cytotoxicity of free ATRA, DBZ, D-LNF, and RD-LNF at a fixed concentration of 25 μg/ml and 50 μg/ml

MTT assay was performed on B16F10 cells after treatment with respective formulations to evaluate the anticancer effect of individual therapeutic agents (Fig. 5a). At first, cytotoxicity of individual free DBZ and ATRA was evaluated. DBZ exhibited a concentration-dependent cytotoxic effect in the melanoma cancer cells while ATRA exhibited a significantly higher cytotoxic effect in the B16F10 cells. At 100 μg/ml, DBZ exhibited a ~ 45% cell viability compared to ~ 22% cell viability for the ATRA indicating the excellent therapeutic efficacy of ATRA. In order to prove the co-delivery of DBZ and ATRA could potentially inhibit the cancer progression, DBZ+ATRA-loaded lipid nanoformulations (RD-LNF) were treated to melanoma cells. DBZ-loaded lipid nanoformulations (D-LNF) containing the single entity drug was used as a reference group to highlight the synergistic efficacy of ATRA (Fig. 5b). As expected, at 25 μg/ml of fixed concentration, ATRA showed slight lower cell viability compared to that of DBZ while nanoparticle-based D-LNF did not improve the anticancer effect of DBZ and remained far from effective. As expected, RD-LNF showed significantly lower cell viability and higher anticancer effect compared to that of D-LNF indicating an apparent synergistic therapeutic effect of dual components towards the melanoma cancer cell death. RD-LNF showed a significantly higher anticancer effect compared to that of any other treatment groups. It might be expected that slow and sustained release of DBZ along with the slow release of ATRA from the nanocarrier system might contribute to the synergistic therapeutic effect. In this study, dacarbazine (DBZ) was the main chemotherapeutic drug and ATRA was employed as a structural component of lipid nanoparticles, therefore did not create a separate group for R-LNF. We have studied the anticancer effect of bare ATRA and DBZ in the cancer cells. Besides, many published reports are proof for the anticancer effect of ATRA; therefore, we have used ATRA as a structural component that could also improve the anticancer efficacy of encapsulated therapeutics in a synergistic manner.

### Colony Formation Assay

**Fig. 6:**
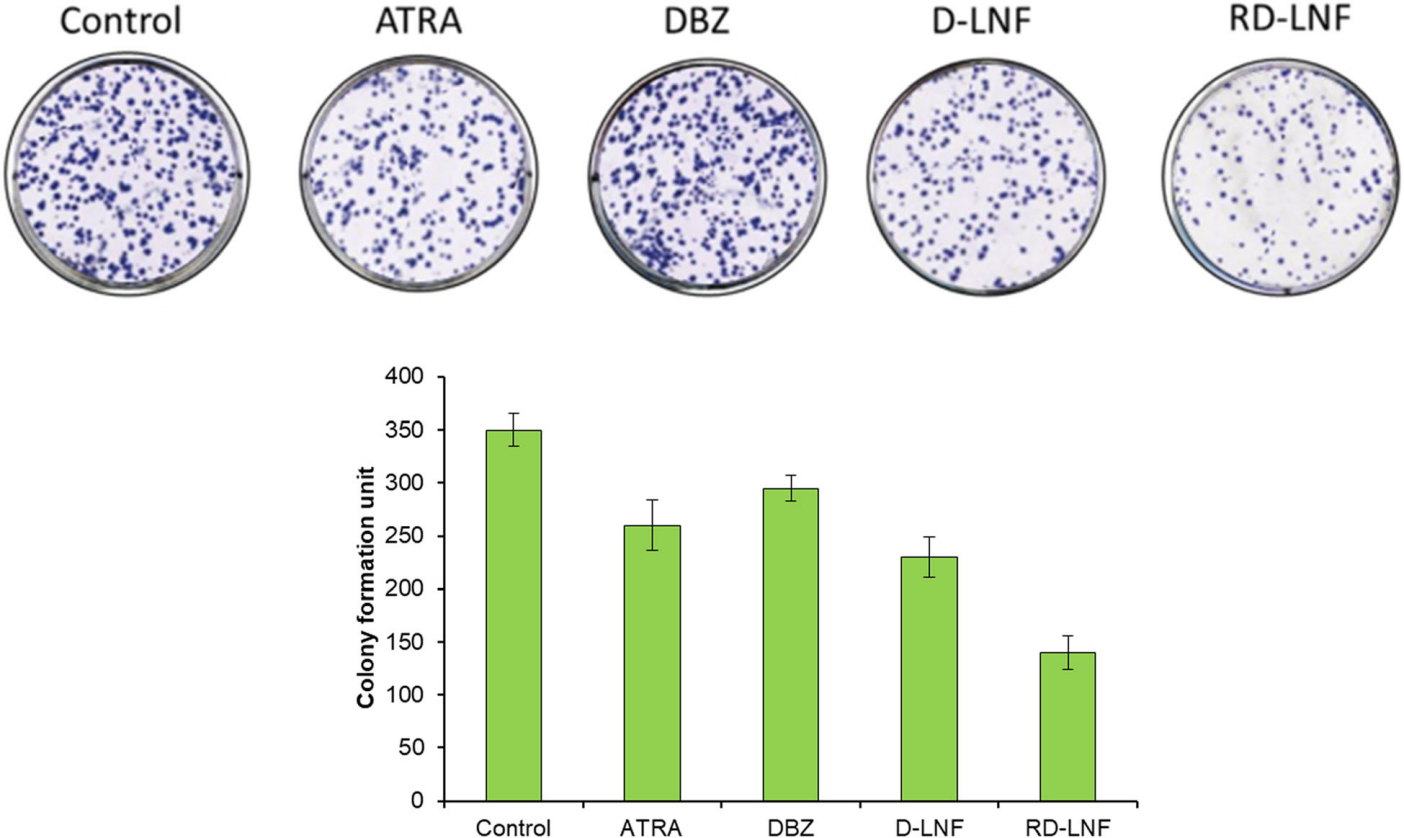
Effect of free ATRA, DBZ, D-LNF, and RD-LNF on the colony formation. B16F10 cells were treated with respective formulations and colony formation was photographed after crystal violet staining

The colony formation assay was performed in order to evaluate the potential of tumorigenesis of melanoma cells. As shown, free DBZ and ATRA have a limited role in inhibiting colony formation and similarly, D-LNF was ineffective in controlling the colony formation (Fig. 6). As expected, the combination of DBZ + ATRA resulted in significant potentiation of inhibition of colony formation compared to that of individual free drugs or single drug-loaded nanoparticles. The colony formulation assay further reiterates the superior anticancer efficacy of RD-LNF over free drugs.

### In Vitro Apoptosis Assay and Cell Cycle Analysis

**Fig. 7:**
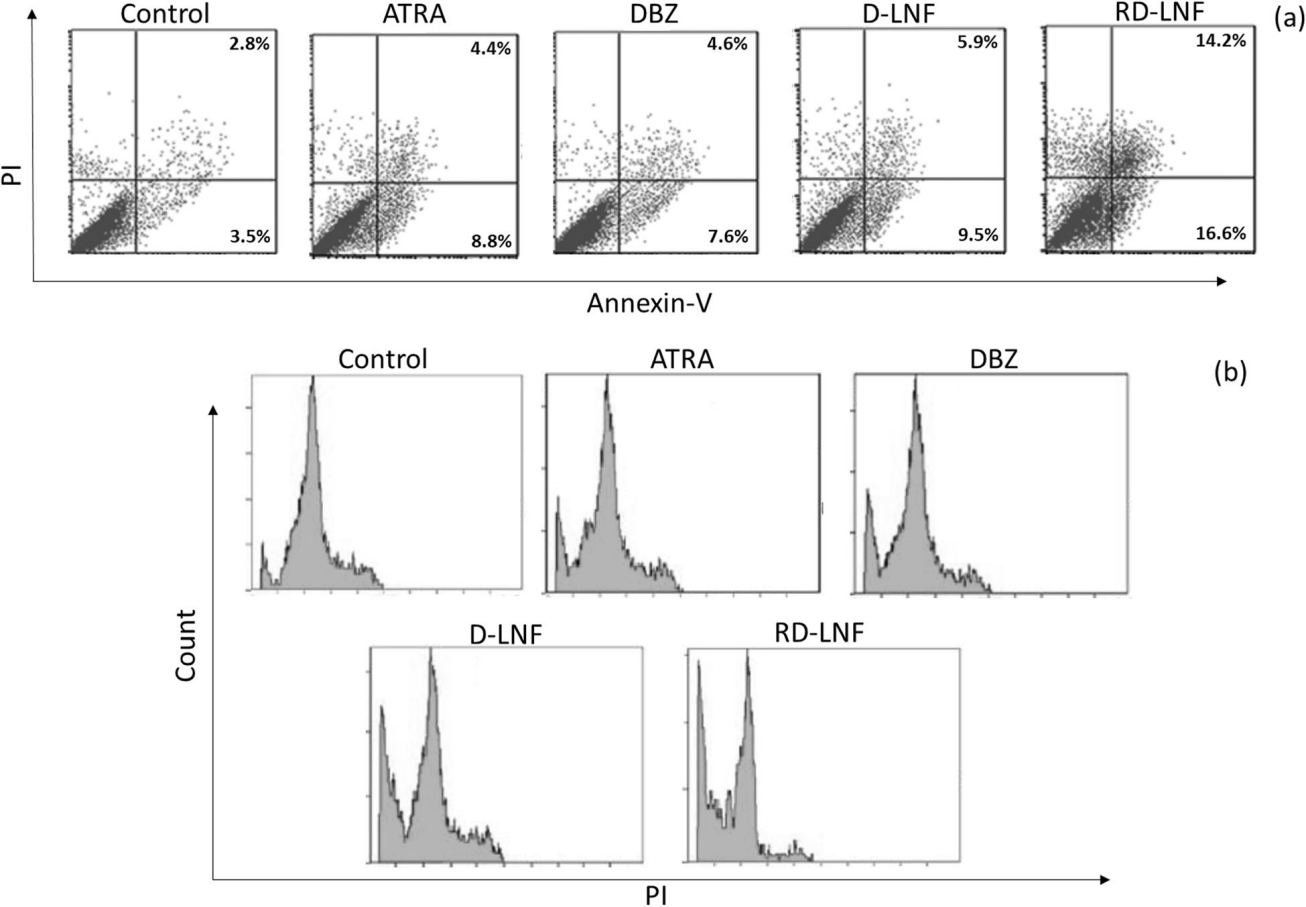
**a** Illustration of apoptosis of B16F10 cells after treatment with free ATRA, DBZ, D-LNF, and RD-LNF. The untreated cells were considered as an appropriate control. The apoptosis was analyzed using flow cytometer. **b** Illustrations of cell cycle analysis performed in B16F10 cells after treatment with respective formulations

The apoptosis induction of B16F10 cells after treatment with D-LNF and RD-LNF was studied using a flow cytometer after staining the cells with Annexin V-FITC/PI mixture (Fig. 7a). As shown, ATRA and DBZ caused only 10–12% of apoptosis of cancer cells and much of the cells remain intact. A slight increase in early apoptotic cells was observed after D-LNF treatment might suggest the effect of the delivery carrier. Importantly, RD-LNF induced a greater proportion of apoptosis cells with a greater proportion of cells in late apoptosis and early apoptosis indicating the superior anticancer efficacy of combinational nanoparticles. RD-LNF showed that late apoptosis cells increased 21.5% while early apoptosis cells increased up to 18.3% and proportion of viable cells decreased from 95 to 52%, respectively, suggesting that combination of DBZ + ATRA contributed to the apoptosis of cancer cells and could inhibit the proliferative inhibition of B16F10 melanoma cells. The apoptosis effect of formulations was further confirmed by cell cycle analysis. As shown, DBZ and ATRA exhibited a slight increase in the sub-G0 population while D-LNF showed a relatively higher sub-G0 population compared to that of individual free drugs (Fig. 7b). Notably, remarkable increase in the sub-G0 population was observed for RD-LNF treated melanoma cells indicating the marked apoptosis of the cancer cells. It is well known that DBZ could increase the p21, caspase-3, and cleaved PARP expression levels and thereby promotes the cell apoptosis through the stabilization of p53. The induction of p53 will inhibit the cell cycle progression [24, 25]. The combination of DBZ + ATRA is expected to potentiate the apoptosis mechanism and halt the cell cycle progression of cancer cells and exhibit the anticancer efficacy.

### Effect of Combinational Nanoparticles on Cell Migration

**Fig. 8:**
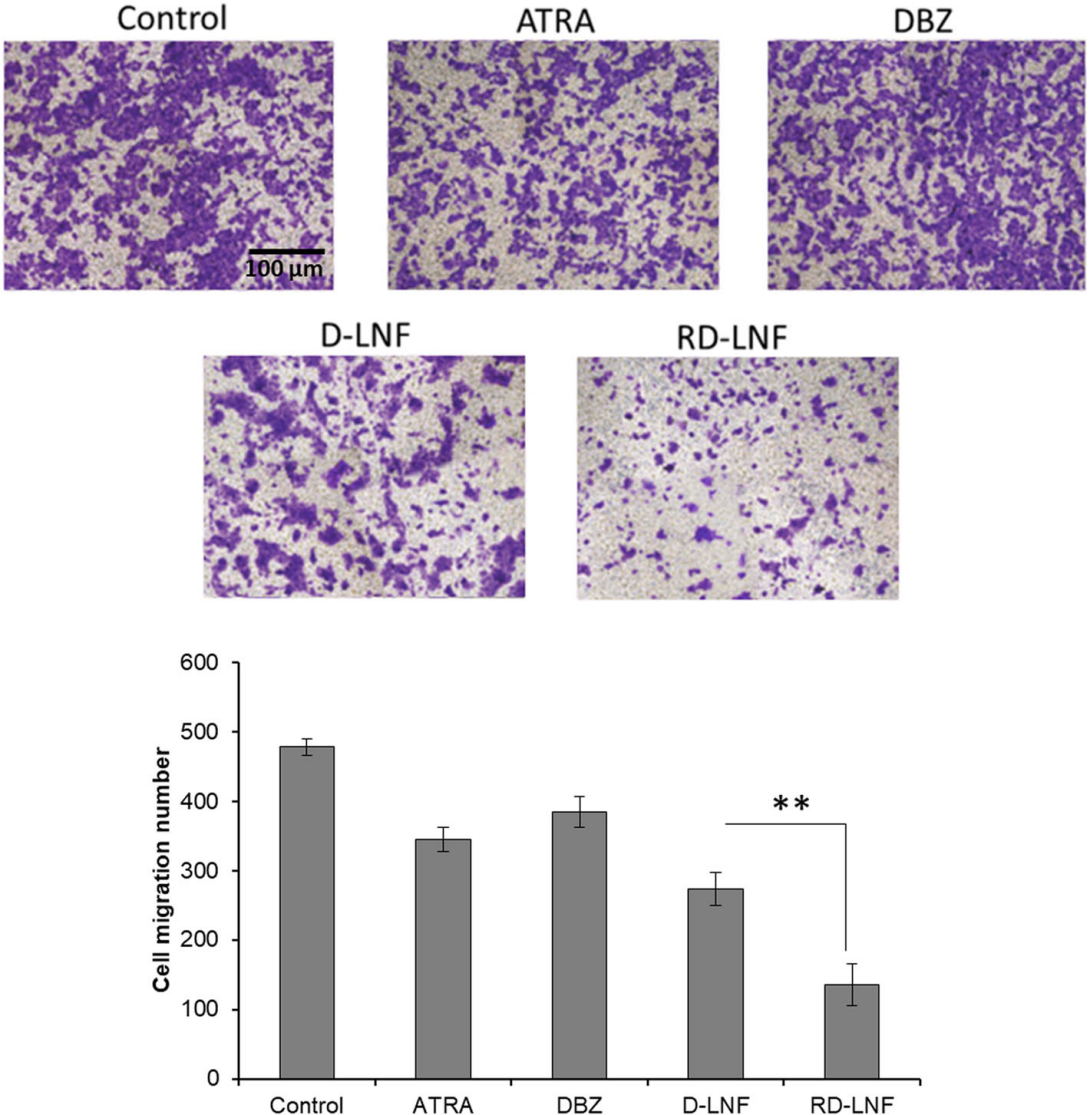
Representative microphotographs of the cell migration assay of B16F10 cells after treatment with free ATRA, DBZ, D-LNF, and RD-LNF and its quantification

The cell migration was analyzed through the transwell membrane and observed the migrating cells by crystal violet staining (Fig. 8). After 24h, control cells had nearly achieved the complete migration of cancer cells. ATRA seems to inhibit cell migration relatively better than DBZ-treated cells. D-LNF also showed a notable inhibitory effect on cell migration; however, the most remarkable cell migration effect was observed in RD-LNF treated B10F16 melanoma cells. As seen, manifold decreased in cell migration was observed compared to that of the non-treated control cells. These results clearly demonstrate that RD-LNF potentially decreases the aberrant malignant cell proliferation and suppress cell migration efficiently.

## Data Availability

Not applicable

## Abbreviations

DBZ: Dacarbazine
ATRA: All-trans retinoic acid
D-LNF: DBZ-loaded lipid nanoformulations
RD-LNF: ATRA/DBZ-loaded lipid nanoformulations
SLN: Solid lipid nanoparticles
